# Human granulocytic anaplasmosis in a Single University Hospital in the Republic of Korea

**DOI:** 10.1038/s41598-021-90327-y

**Published:** 2021-05-25

**Authors:** Da Young Kim, Jun-Won Seo, Na Ra Yun, Choon-Mee Kim, Dong-Min Kim

**Affiliations:** 1 Department of Internal Medicine, Chosun University College of Medicine, 588 Seosuk-dong, Dong-gu, Gwangju, 501-717 South Korea; 2 Department of Premedical Science, Chosun University College of Medicine, 588 Seosuk-dong, Dong-gu, Gwangju, 501-717 South Korea; 3 Department of Internal Medicine, School of Medicine, Chosun University, 365, Pilmun-daero, Dong-gu, Gwangju Metropolitan City, 61453 Republic of Korea

**Keywords:** Diseases, Medical research

## Abstract

To date, only a few studies have analyzed the clinical characteristics and genetic features of human granulocytic anaplasmosis (HGA) in South Korea. Thus, in this study, we investigated the clinical characteristics of HGA and methods used for clinical diagnosis. The clinical characteristics of patients with HGA were studied retrospectively. We reviewed the medical charts of 21 confirmed patients with HGA admitted to the Chosun University Hospital, located in Gwangju, South Korea. Twenty-one HGA patients visited the hospital 2–30 days (median 7 days) after the onset of symptoms. Fourteen patients (66.7%) had fever, which was alleviated 2 h (range 0–12.75 h) after starting treatment with doxycycline. Of the 18 patients who underwent peripheral blood (PB) smear test, only one (5.6%) had morulae. Additionally, only 4/17 patients (23.5%) had morulae in the PB smear reconducted after the confirmation of anaplasmosis. All 21 patients recovered without significant complications. As per results of the blood tests conducted at the time of admission, 7/21 (33.3%) and 5/21 (23.8%) patients showed at least 1:16 and 1:80 of IgM and IgG titers, respectively. Most HGA patients in Korea recovered without significant complications. The indirect immunofluorescence antibody diagnosis or morulae identification for HGA in this study had low sensitivity in the early stage of the disease.

## Introduction

Human granulocytic anaplasmosis (HGA) is an acute tick-borne febrile illness caused by *Anaplasma phagocytophilum*^1^.

The first report of human infection in the United States was in 1994, and there were 6.3 incidences of HGA per million people between 2008 and 2012^2^. The vectors for HGA are hard ticks; *Ixodes scapularis* and *I. ricinus* are known to be the major vectors in the northwest, north, and midwest of the United States, and in Western Europe^3^.

In South Korea, *A. phagocytophilum* has been found in the ticks *Haemaphysalis longicornis, I. nipponensis*, and *I. persulcatus*^4,5^, and was first reported in 2014^6^. However, until now, there have been few studies on the clinical characteristics and genetic features of HGA in South Korea, or on the clinical usefulness of immunofluorescence antibody (IFA) for its diagnosis. We investigated the clinical characteristics of HGA and the diagnostic methods used to confirm HGA in patients.

## Methods

### Study approval

This study was approved by the institutional review boards (IRB) of Chosun University Hospital (IRB no. 2013-10-001). Written informed consent was obtained from all patients or their legal guardians in accordance with the Helsinki Declaration. All methods were performed in accordance with relevant Korean guidelines and regulations.

### Patients and study site

Chosun University Hospital, an 849-bed hospital, is located in Gwangju, South Korea. We performed IFA and polymerase chain reaction (PCR) tests for *A. phagocytophilum* in patients admitted to this hospital with a history of tick bite or outdoor activity and with fever between 2013 and 2018. We reviewed the medical charts of 21 confirmed HGA patients.

### Diagnostic methods

A diagnosis of HGA requires one of the following criteria^7^: (1) successful culture of *A. phagocytophilum*, (2) a four-fold elevation of antibody titers for *A. phagocytophilum* in the acute phase compared with the recovery phase, (3) positive PCR results for two or more target genes, or (4) a positive PCR, for at least one target gene and at least 1:16 and 1:80 IgM and IgG antibody titers, respectively, for *A. phagocytophilum.*

IFA antibody tests (Fuller Laboratories, Fullerton, CA, USA) for *A. phagocytophilum* were performed at the Korea Centers for Disease Control and Prevention (KCDC) following the manufacturer’s instructions, as previously described^1^. The serological positive cut-off values used were 1:80 and 1:16 for IgG and IgM, respectively.

### Genetic analysis

Conventional and nested PCR tests were performed in our hospital using buffy coats and tick bite sites, targeting genes specific for *Ehrlichia* or *Anaplasma*, including the GroEL heat-shock protein gene (*groEL*), the 16S ribosomal RNA gene (*16S rRNA*), the ankyrin-repeat protein AnkA gene (*ankA*), and the major surface protein 2 gene (*msp2*)^1^. PCR was performed using AmpliTaq Gold 360 Master Mix (Applied Biosystems, Foster City, CA, USA) and a Veriti96-Well Thermal Cycler (Applied Biosystems). PCR was performed with a total of 20 µL of reaction solution consisting of 1 μL of 5 μM forward primer, 1 μL of reverse primer, 10 μL of Master mix, 2 μL of GC enhancer, and 4 μL of distilled water.

Nested PCR was performed using the same reaction mixture as that used for conventional PCR. For the nested PCR, however, the first PCR product was used as the template DNA, instead of genomic DNA, along with nested PCR primers. Subsequently, the PCR product was subjected to electrophoresis on a 1.2% agarose gel containing ethidium bromide. The positive PCR products were purified using the QIAquick Gel Extraction Kits (QIAGEN, Hilden, Germany), and the DNA was sequenced using Solgent (Daejeon, South Korea). Sequencing was performed from both ends with the primers used in the nested PCR in an automatic sequencer (ABI Prism 3730XL DNA analyzer, Applied Biosystems). The sequencing data were analyzed using the BLAST network service (Version 2.33; www.technelysium.com.au/chromas.html) of the National Center for Biotechnology Information (National Institutes of Health). Complete DNA sequences were used for the construction of phylogenetic trees, for which a 1000 times bootstrap analysis was applied to increase reliability^1^.

### Statistical analysis

All data were analyzed using SPSS, Version 18.0 (SPSS Inc., Chicago, IL, USA). The continuous and categorical variables are presented as median ± interquartile range (IQR) and percentage, respectively. The Wilcoxon signed-rank, Friedman, and Mann–Whitney U tests were used to compare mean values of the white blood cell (WBC) counts performed on the admission day, and 1 and 2 weeks after admission. *P* values < 0.05 were considered to be statistically significant.

## Results

### Diagnostic tests

Among the patients who visited our hospital between 2013 and 2018, PCR tests were performed on 789 patients who were suspected of having tick-borne infectious diseases, such as anaplasmosis, Lyme disease, tsutsugamushi disease, severe fever with thrombocytopenia syndrome, *Bartonella* infection, Q fever, or *Borrelia* infectious diseases, and an IFA test was performed for 375 patients. Of these, 18 patients had positive results in PCR tests targeting two or more genes for *A. phagocytophilum*. An IFA test for anaplasmosis was performed in 154 of the patients, and 15 patients showed a four-fold increase in antibody titer between the acute and recovery phases. Of these, 14 patients showed double-positivity by PCR and IFA tests. Twenty-four patients out of 139 had an IgM titer of 1:16 or higher, or an IgG titer of 1:80 or higher in the acute phase, but there was no increase in antibody levels in the recovery phase. In addition, when PCR for *Anaplasma*-specific target genes was negative, the cases were diagnosed as other diseases, which indicate the existence of false positives or past infections. A total of 21 patients were confirmed to have HGA.

### Clinical characteristics

**Figure 1:**
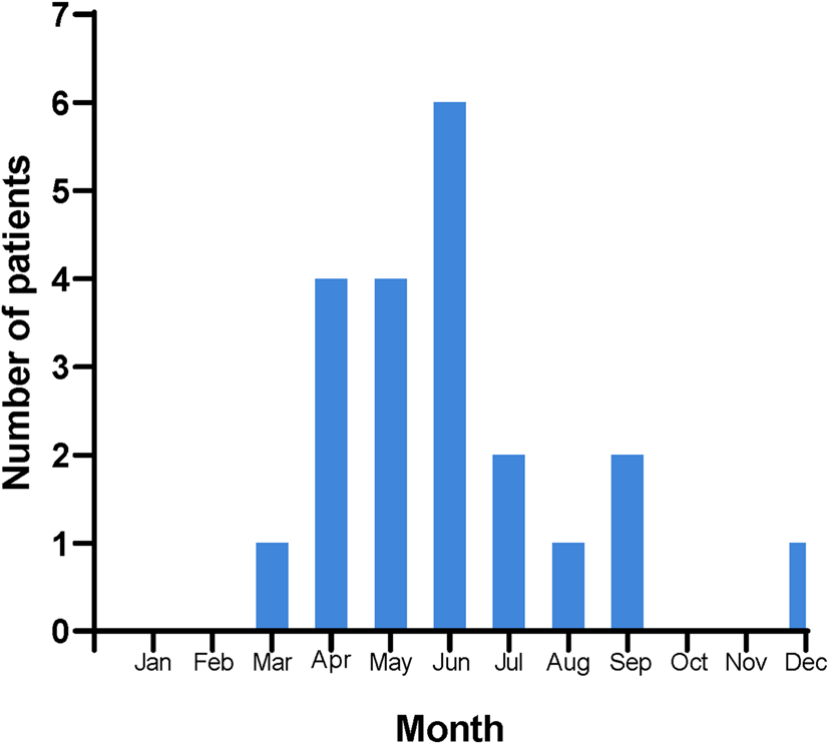
Anaplasmosis cases by month of symptom onset. Twenty HGA patients were diagnosed in our hospital from March to September.

These 21 patients visited the hospital 2–30 days (median 7 days) after the onset of symptoms (Table 1). Twenty patients had symptom onset in March–September and one patient in December (Fig. 1).

**Table 1:** Patient characteristics, clinical manifestations, and lab findings.

Characteristics	Value
Median age, years (range)	70.7(49–83)
Male (%)	7(33.3)
Farmer (%)	16(76.2)
Duration 1, days (range)	7(6–13)
History of tick bite (patient/physician) (%)	10(47.6)/9(42.8)
Intensive care unit admission (%)	4(19.0)
Fatal course (%)	0(0)

Signs or symptoms	No. (%)
Fever (temperature > 37.5 °C)	14(66.7)
Gastrointestinal symptoms	11(52.4)
Nausea/vomiting	9(42.9)
Diarrhea	6(28.6)
Abdominal pain	2(9.5)
Dyspepsia	2(9.5)
Chills	12(57.1)
Myalgia	8(38.1)
Cough	5(23.8)
Headache	3(14.3)
Mental changes	3(14.3)

Laboratory findings	No. (%)
Leukopenia (< 5000/µL)	18(90.0)
Thrombocytopenia (< 150,000/µL)	18(90.0)
Elevated AST or ALT (> 40 U/L)	17(81.0)
Elevated CRP (> 0.3 mg/dL)	18(90.0)
Morulae, first/reinterpretation	1(1/20, 5%)/4(4/17, 23.5%)

*AST* aspartate aminotransferase, *ALT* alanine aminotransferase, *CRP* C-reactive protein, *No* number.

Duration 1 is the days from the symptom onset to the initial visit (days).

History of tick bite means tick bite site cognition by patient or physician.

Laboratory findings are reported with the samples on the admission day.

Morulae initially checked as a reported paper by the first interpretation and reviewed using peripheral blood smear slides for reinterpretation.

The mean age of the patients was 70.7 years (range 49–83 years). Seven patients were males (33.3%) and 14 were females (66.7%). Among the 21 patients, one had a history of tsutsugamushi disease.

Of the 21 patients, 10 (47.6%) were aware of having experienced tick bites, whereas nine (42.8%) had lesions that were suspected to be tick bites upon physical examination, although the patients were unaware of any exposure of ticks. No suspected tick bite lesions were found in the remaining two patients (9.5%).

Fourteen patients (66.7%) had a fever, with temperature above 37.5 °C (37.8–39.4 °C); seven had fever alleviation before antibiotic administration, whereas the other seven had temperature reduction at a median of 2 h (range 0–12.75 h) after doxycycline treatment. Their fever mostly subsided within 24 h.

Eleven patients (52.4%) had gastrointestinal symptoms and eight (38.1%) had myalgia. Among the 21 patients, four (19.0%) required intensive care treatment after hospitalization, due to low blood pressure or high oxygen demand but there was no mortality.

The patients were treated either with concurrent ceftriaxone and doxycycline or doxycycline monotherapy (Supplementary Table 1).

### Laboratory findings

The results of the tests performed on the day of admission showed that 13 patients (65%) had leukopenia and 18 patients (90.0%) thrombocytopenia, and high levels of aspartate aminotransferase (AST), alanine aminotransferase (ALT), and C-reactive protein (CRP) were detected in 18 (90.0%), 17 (81.0%), and 18 (90.0%) patients, respectively (Supplementary Fig. 1a–d).

Peripheral blood (PB) smears were performed for 18 patients, and only one patient (5.6%) was found to have morulae. Two specialists in diagnostic medicine reviewed the PB smears of the 17 patients with anaplasmosis retrospectively, after the diagnosis. Each of the doctors found morulae in four patients (23.5%) (Supplementary Tables 2 and 3).

### Genetic and serological tests

Antibody titer follow-up was performed at the time of visit and at week 2 and week 4. We performed a follow-up antibody test for more than 2 weeks for 19 of 21 patients. Twelve patients were followed-up for more than 4 weeks, and six patients were followed-up for at least 8 weeks. Excluding one patient who was not tested in the follow-up, 15/20 (75.0%) patients showed an increase in antibody titer by least four times compared to those measured on the day of admission.

**Figure 2:**
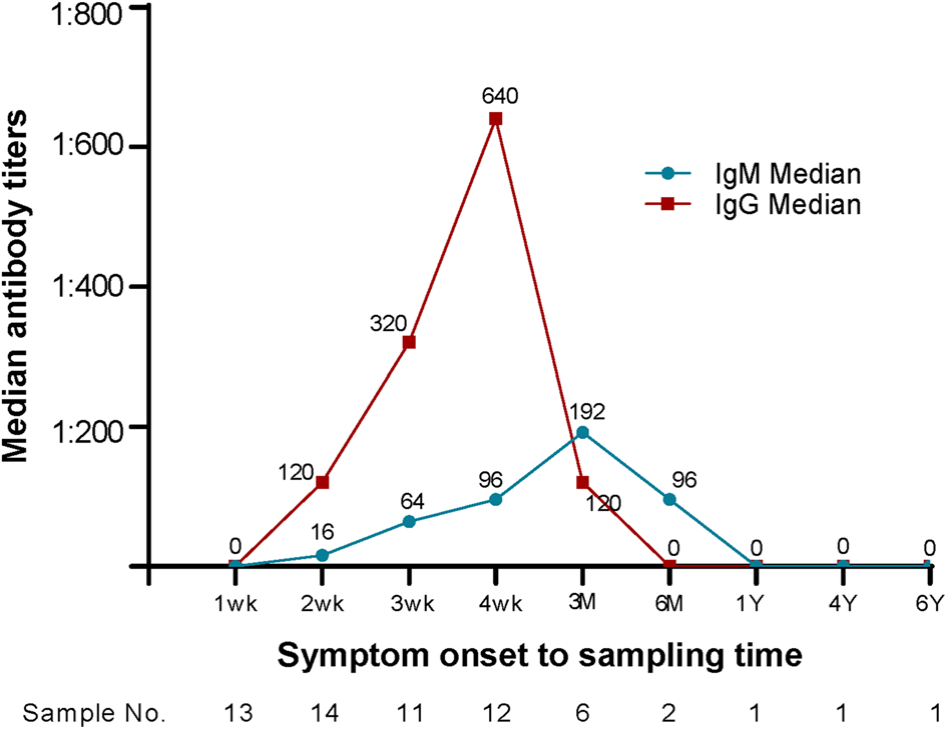
Median antibody titers by symptom onset to sampling time. In the follow-up test on antibody titer initiated from the onset of symptoms, IgG antibody titers increased more rapidly than those of IgM. IgG reached the peak 4 weeks after the onset of symptoms, followed by a decline, and there was no detectable antibody titer after 3 months. IgM reached a peak 3 months from the onset of symptoms, followed by a decline, and there was no detectable antibody titer 1, 4, and 5 years after symptom onset.

IgG antibody titers increased more rapidly than those of IgM. IgG titers reached a peak 4 weeks after the onset of symptoms and then declined. After 3 months, there was no detectable antibody titer. IgM titers reached a peak 3 months after the onset of symptoms, followed by a gradual decline. There was no detectable antibody titer in the follow-up at 1, 4, and 5 years after the onset of symptoms (Fig. 2).

When the IgM antibody titer cut-off was set at 1:16 or higher, 79% of patients were positive 2 weeks after the onset of symptoms, a observation that persisted for 6 months.

**Figure 3:**
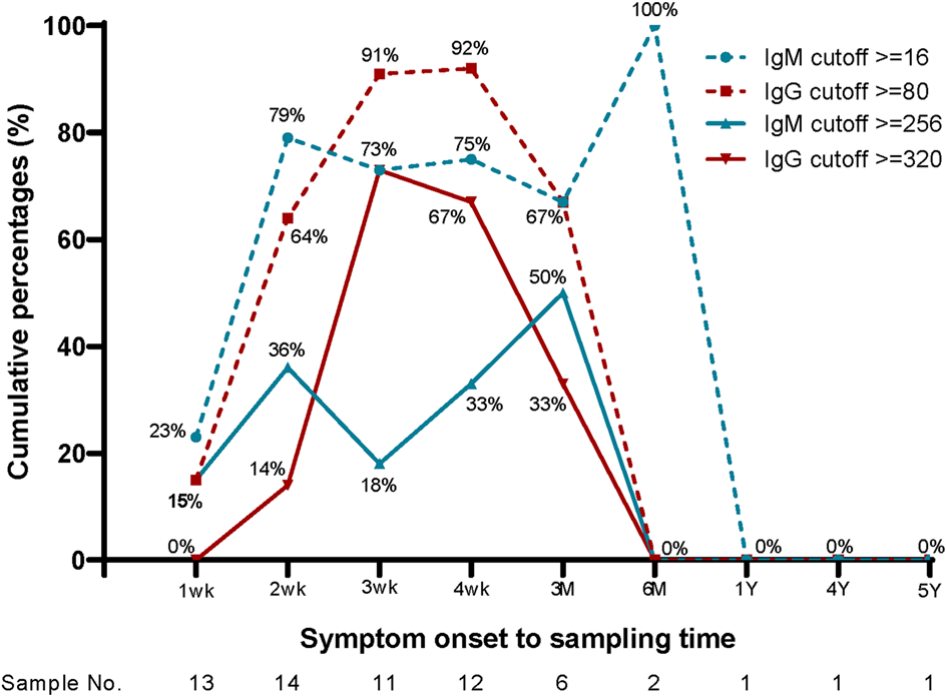
The cumulative percentage for positive rate by symptom onset to sampling time. When a 1:16 or higher IgM titer was set as the cut-off, 79% of the patients were positive 2 weeks after the onset of symptoms and remained positive until the sixth month from the onset of symptoms. When a 1:80 or higher IgG titer was set as the cut-off, up to 92% of the patients were positive 4 weeks after the onset of symptoms.

When the IgG antibody titer cut-off was set at 1:80 or higher, up to 92% were positive 4 weeks after the onset of symptoms, which lasted for 3 months. Even when the antibody titer cut-off values were set at 1:256 for IgM or 1:320 for IgG, they were positive for 3 months after the onset of symptoms (Fig. 3).

A follow-up test of antibody titer was also performed based on the day of the onset of symptoms (Supplementary Table 4).

PCR was performed using blood samples drawn on the day of admission targeting *groEL*, *ankA*, and *16S rRNA*; eighteen patients (85.7%) were found to be positive for two or more genes. The PCR tests targeting *groEL*, *ankA*, and *16S rRNA* were positive in 19 (90.5%), 19 (90.5%), and 17 (81.0%) patients, respectively. Phylogenetic trees were constructed to identify clusters (Supplementary Fig. 2a–c).

The nested PCR for *16S rRNA* and its DNA sequence analysis found a sequence similarity of 99.8–100% among all the patients, and the sequence was also found to be identical (99.9–100%) to that of *A. phagocytophilum* (Genbank accession no. CP000235, CP006616), which was identified in a patient from the United States. In the phylogenetic tree created using the 837 bp rRNA, all of the samples from the patients formed a cluster with *A. phagocytophilum*. The nested PCR for *groEL* and its DNA sequence analysis had a 97.6–100% sequence similarity among all the patients, and the sequence was identical to that of *A. phagocytophilum* (Genbank accession no. KU519285, KU519286, KU519287) identified from cats and dogs in South Korea. The nested PCR for *ankA* and its DNA sequence analysis found a 95.2–100% sequence similarity among all the patients, and the sequence was identical to that of *A. phagocytophilum* (Genbank accession no. KJ677106, KT986059) identified from a patient in South Korea. Taken together, the DNA sequence analyses of these PCR products confirmed that the 21 patients harbored *A. phagocytophilum* (Supplementary Fig. 2a–c).

## Discussion

Since the first report of an HGA patient in South Korea in 2014^6^, only case reports related to the diagnosis of HGA^8,9^ and papers related to co-infection with other tick-borne diseases have been published in South Korea^10,11^. However, no systematic studies have been conducted to assess its clinical characteristics, and most of the studies which do exist are case reports. Among the patients who were admitted to one university hospital, 21 patients were diagnosed with HGA. This result suggests the possibility of under-diagnosis of this emerging infectious disease.

One study in the US^12^ analyzed the clinical manifestations and lab results of 44 patients who were confirmed to have HGA based on blood cultures, and 39 (88.6%), 37 (84.1%), and 36 (81.8%) patients complained of fever/cold sweat, fatigue, and headache, respectively. Another study in China^13^ investigated the epidemiological and clinical characteristics of HGA in 83 patients. The most common clinical manifestations were fever, weakness, and anorexia in 83 (100%), 71 (85.5%), and 64 (77.1%) patients, respectively. In contrast, a relatively low number of patients (14 patients, 66.7%) had a fever, while 11 (52.4%) and 8 (38.1%) patients had gastrointestinal symptoms and myalgia, respectively in our study. As patients complained of relatively nonspecific symptoms, the diagnosis was difficult, unless patients were tested for HGA infection. However, once patients were treated with antibiotics following diagnosis with HGA, most of them showed alleviation of the fever within 24 h, and all of them recovered without critical complications, whereas another study^13^ showed a 26.5% mortality and severe complications.

Regarding the hematological tests carried out on the day of admission, the results (except for one patient who was not tested for complete blood count) showed thrombocytopenia, as shown by a 5000/μL or lower WBC count and a 150,000/μL or lower platelet count in 18 (90.0%) patients. In addition, 17 (81.0%) patients had an elevation of AST or ALT (over 40 U/L), and 18 (85.7%) patients showed an increase in CRP level of at least 0.3 mg/dL.

Dumler^14^ reported that a blood smear within 1 or 2 weeks from the onset of the disease could identify morulae in 25–75% and 63% of HGA patients, respectively. In another study^12^, 34 (77.3%) patients were found to have HGA morulae. In contrast, of the 21 confirmed patients in our study, 18 patients were subjected to PB smear and only one patient (5.6%) was found to have a morula. Since there were considerable differences from previous reports, two specialists in diagnostic medicine rereviewed the stored slides of the 17 patients with anaplasmosis and found morulae in four (23.5%) patients. In summary, there was a small number of patients with morulae compared with previously reported numbers, suggesting that it could be difficult for suspected anaplasmosis patients to be confirmed on the basis of morula identification in South Korea.

Silaghi et al.^15^ showed that bacteria can be detected successfully only when blood is smeared immediately after its collection. In the case of leukocytotropic species (*A. bovis* and *A. phagocytophilum*), there may be few infected WBCs due to leukopenia, and in the case of granulocytotropic species, morulae can be distributed unevenly and sparingly, suggesting that at least 800–1000 granulocytes should be tested.

Therefore, it cannot be concluded that a negative result from the blood smear is an indication of the absence of anaplasmosis, and additional laboratory diagnostic tests should always be performed, as Howell–Jolly bodies, other inclusions, contaminant particles or platelets, and nuclear fragments can also be judged as false positives^15^.

In a study on the clinical and laboratory characteristics of human granulocytic ehrlichiosis (HGE)^16^, about 37 patients (90%) had a history of exposure to ticks. In a study on HGE patients in medical centers in New York^17^, 13/18 patients (72%) had tick bites. In this study, only 10/21 patients (47.6%) were aware of tick bites within a month before admission, whereas nine patients (42.8%) had lesions suspected to be tick bite sites, during a physical examination by medical staff, although they were unaware of the bites.

A clinical serological follow-up study of 30 HGA confirmed patients in Slovenia^18^ identified seroconversion in three (10%) patients through an antibody titer test, and an elevation of antibody titer of more than four times in 25 (83.3%) patients. A reciprocal titer of 128 or higher was deemed positive. In follow-up tests, a 128 or higher IFA titer was found in 14/29 patients (48.3%), 17/30 (56.7%), 13/30 (43.3%), and 12/30 (40.0%) patients in the 6th, 12th, 18th, and 24th months after onset of symptoms, respectively.

In another study^19^, most patients were positive for the antibody within 2 weeks after the onset of symptoms and reached a peak antibody titer after a month. Approximately 50% of the patients had an antibody titer of 80 or higher until the 12th month after treatment. In the present study, the HGA patients showed an increase in IgM antibody titer from the onset of symptoms until the third month, on average, and then a decrease, and these antibodies became undetectable after the sixth month. The IgG antibody titer rapidly increased until the fourth week, on average, followed by a decline, and these antibodies then became undetectable after the third month.

There was a considerable difference in antibody titer compared to previous reports, and the underlying reason is unclear. In another study^19^, NY-13, a local strain in the US, was used for the preparation of antigen slides, whereas in this study, the *A. phagocytophilum* strain (NCH-1) cultured from the HL-60 cell line from Fuller Laboratories was used for IFA slide preparation, instead of a local human isolate, followed by tests at the KCDC. Therefore, the antibody titer difference might be attributable to the differences in antigens, an issue which needs to be further investigated.

According to a study on HGA patients in Japan^20^, differences were found in the major surface antigen proteins, depending on the cell line (THP-1 and HL60) used for IFA and western blotting in HGA diagnosis. Therefore, it was reported that the test results could be different depending on the cultured cell line.

In another previous study^19^, most of the patients exhibited seroconversion, although two patients failed to form an antibody titer; one of them was treated 1 day after the onset of symptoms. Another two patients were treated with antibiotics 1 day after the onset of symptoms, although antibody titers were produced.

In a follow-up study of IFA titers in HGE patients in the US^21^, the mean antibody titer and time of early, late, or no administration of doxycycline groups were compared. When an IFA of 80 or higher was considered positive, 47% of the patients showed positive results in antibody titer in the early stage of symptom onset, and 71/74 (96%) patients were positive in the follow-up in the fourth week. The mean IFA titers were 954, 1613, and 880 in the early, late, and no doxycycline administration groups, respectively. Doxycycline administration during the first week of symptom onset did not affect antibody formation. However, serum antibodies disappeared earlier in those with early administration of doxycycline than in those with late or no administration. However, for the confirmed patients in our study, when an IgM antibody titer of 16 or higher or an IgG titer of 80 or higher was detected, the median values for the length of time until the formation of antibody titer in patients with doxycycline administration within 3 days, 4–7 days, and 90–30 days from symptom onset were 17 (IQR, 12–19.5), 20 (IQR, 11–72), and 37 days (IQR, 21–68.5), respectively. This observation indicates that early administration of antibiotics tended to lead to the early formation of antibodies; this difference should be further studied.

Among the confirmed patients in this study, 75% had an IgM titer of 1:16 or higher and 91.7% had an IgG titer of 1:80 or higher 4 weeks after symptom onset.

Only 70% of the patients showed four times or higher IgM or IgG titers compared with the titers of tests performed on admission day.

There are currently no commercially available diagnostic tests that can be used to differentiate between past and recurrent infections of *A. phagocytophilum.* However, in the case of patients 4 and 13, who presented with high antibody titers in the acute phase and overall high IgG titers, the possibility of reinfection cannot be ruled out.

A limitation of this study is its small sample size (only 21 patients) and single-center nature. Another limitation of the study is that long-term follow-up tests for more than 6 months were performed in only 2 out of 21 patients, making it difficult to find statistical significance.

Therefore, a larger number of patients will need to be enrolled for additional studies on the clinical characteristics of anaplasmosis, prognostic factors, and features affecting the mortality rate.

In conclusion, most patients with HGA in South Korea recovered without significant complications. The indirect IFA diagnosis using an *A. phagocytophilum* strain (NCH-1) cultured from the HL-60 cell line or morulae identification for HGA had low sensitivity in the early stage of the disease. HGA is not infrequent in Korea. A high index of suspicion is warranted because the sensitivity of the initial test (mainly PB smear) is low, and the diagnostic accuracy is limited. Our study suggests that HGA is underdiagnosed in Korea, considering that only 21 patients were diagnosed with the disease in a single university hospital for 5 years.

Patients with *Anaplasma* have relatively low IFA titer at the beginning of symptom onset, so PCR tests could be used for early diagnosis. Moreover, a diagnosis of anaplasmosis cannot be excluded based on the absence of morulae in blood smears.

## Supplementary Information

Supplementary Figure Legends.

Supplementary Figure 1.

Supplementary Figure 2a.

Supplementary Figure 2b.

Supplementary Figure 2c.

Supplementary Tables.

## Data Availability

Data and materials are available upon request to the corresponding author.
