# Supplementary material for: Human granulocytic anaplasmosis in a Single University Hospital in the Republic of Korea
Source: Sci Rep. 2021 May 25;11:10860. doi: 10.1038/s41598-021-90327-y (PMC8149831; doi:10.1038/s41598-021-90327-y)
Supplement: Supplementary file 1 — Supplementary Figure Legends. [file 41598_2021_90327_MOESM1_ESM.doc]

**Supplementary Figure 1. Significant changes in the lab tests by sampling time**

1a. The mean values of WBC, ANC, and ALC are presented.

1b. Seventeen out of 21 (81.0%) patients had an elevation in AST or ALT on the admission day, corresponding to the peak values, which tended to decline in the first and second weeks after admission.

1c. The PLT was at the lowest on the admission day, and then gradually increased.

1d. Eighteen out of 21 (90.0%) patients showed an increase in CRP.

The Wilcoxon signed-rank, Friedman, and Mann-Whitney U tests were used to compare mean values of laboratory tests conducted on the admission day and 1 and 2 weeks after admission.

A: The day of admission

B: 1 week from admission

C: 2 weeks from admission

WBC, white blood cell; ANC, absolute neutrophil count; ALC, absolute lymphocyte count; No, number; AST, aspartate aminotransferase; ALT, alanine aminotransferase; PLT, platelet; CRP, C-reactive protein

**Supplementary Figure 2a. Phylogenetic trees based on sequences of the *16S rRNA* gene**

**Supplementary Figure 2b. Phylogenetic trees based on sequences of the *groEL* gene**

**Supplementary Figure 2c. Phylogenetic trees based on sequences of the *ankA* gene**
