# Supplementary figures and images for: Human granulocytic anaplasmosis in a Single University Hospital in the Republic of Korea
Source: Sci Rep. 2021 May 25;11:10860. doi: 10.1038/s41598-021-90327-y (PMC8149831; doi:10.1038/s41598-021-90327-y)

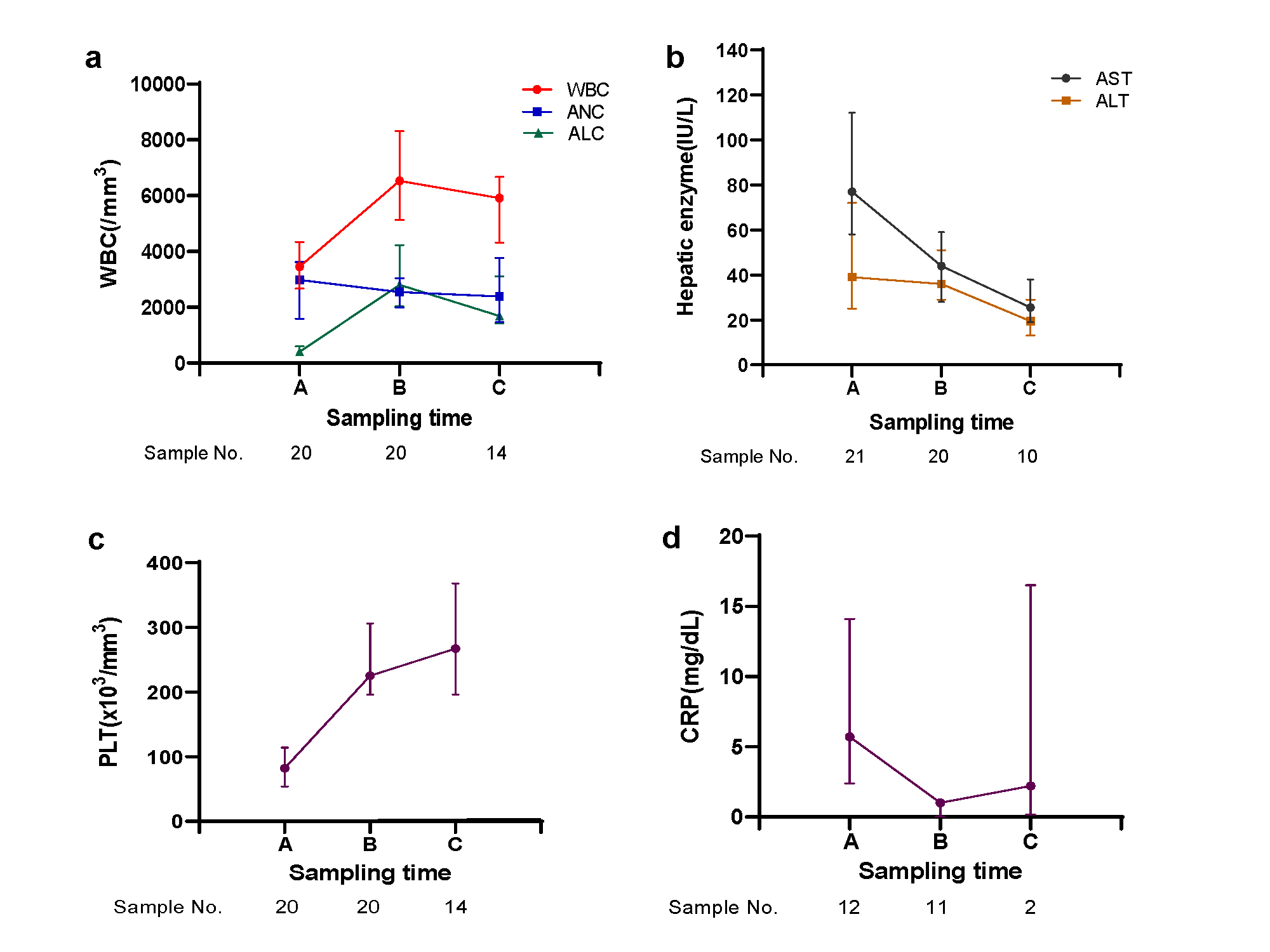

Supplement: Supplementary file 2 — Supplementary Figure 1. [file 41598_2021_90327_MOESM2_ESM.tiff]

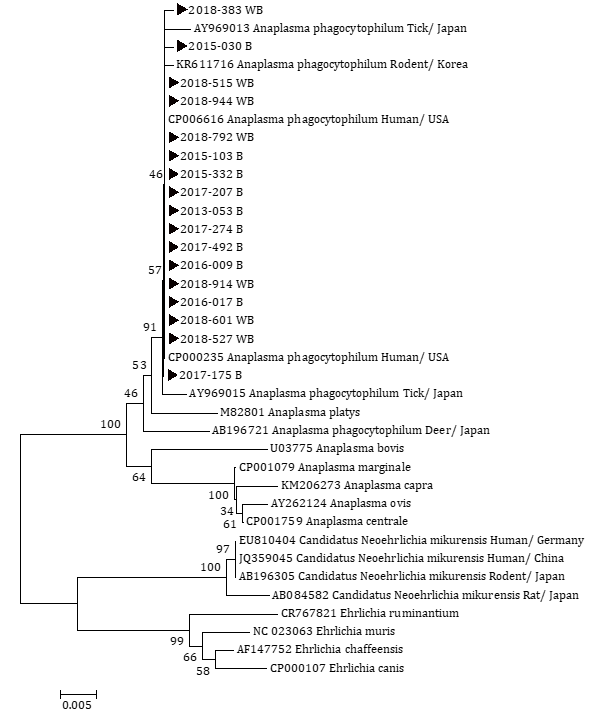

Supplement: Supplementary file 3 — Supplementary Figure 2a. [file 41598_2021_90327_MOESM3_ESM.tif]

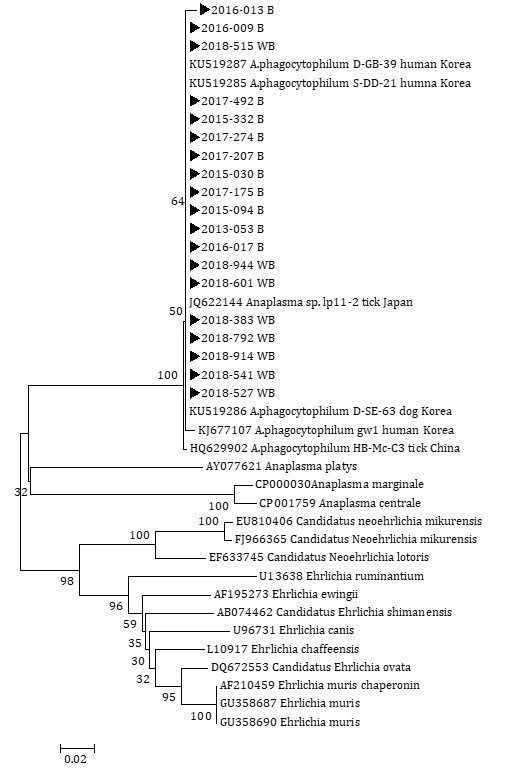

Supplement: Supplementary file 4 — Supplementary Figure 2b. [file 41598_2021_90327_MOESM4_ESM.tif]

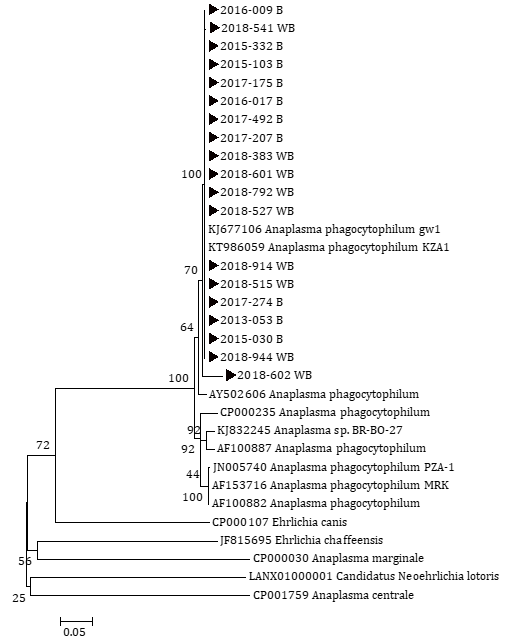

Supplement: Supplementary file 5 — Supplementary Figure 2c. [file 41598_2021_90327_MOESM5_ESM.tif]
