# Supplementary material for: Human granulocytic anaplasmosis in a Single University Hospital in the Republic of Korea
Source: Sci Rep. 2021 May 25;11:10860. doi: 10.1038/s41598-021-90327-y (PMC8149831; doi:10.1038/s41598-021-90327-y)
Supplement: Supplementary file 6 — Supplementary Tables. [file 41598_2021_90327_MOESM6_ESM.docx]

**Supplementary data**

**Supplementary Table 1. Clinical manifestations and treatment**

| No | Age/  Sex | Month/  Year | Duration 1^¶^  (Day) | APACHE | Peak  fever  (℃) | Sign and Symptoms | | | | | | Tick bite site  Cognition  by  Patient  /Physician | Treatment  (Dose period) | Duration 2^*^  (Hours) | Duration 3^†^  (Hours) |
| --- | --- | --- | --- | --- | --- | --- | --- | --- | --- | --- | --- | --- | --- | --- | --- |
|  |  |  |  |  |  | Chills | N/V | Myalgia | Cough | Diarrhea | Others |  |  |  |  |
|  |  |  |  |  |  |  |  |  |  |  |  |  |  |  |  |
| 1 | 79/M | Apr-15 | NA | NA | 39.0 | + | - | + | + | - | Hyperemic conjunctiva  Febrile sensation | Lt. axillar  (Physician) | Cef(7)→Doxy(10) | 3 | 0 |
| 2 | 72/M | Apr-16 | UK | 12 | 38.1 | + | - | + | + | - | Epigastric discomfort | - | Cef+Doxy(5)  →Doxy(5) | 2 | 0 |
| 3 | 73/F | Mar-16 | 13 | 10 | 38.6 | + | + | - | - | - | Headache,  Sore throat,  Abdominal pain | Back  (Patient) | Doxy+Fluco(6)  →Fluco(20) | 2 | 10 |
| 4 | 75/F | Apr-16 | 10 | 11 | 39.2 | + | + | + | - | - | Abdominal pain | Buttock  (Patient) | Cef+Doxy(4)  →Doxy(6) | 0 | 4 |
| 5 | 71/F | Jun-15 | UK | NA | 39.2 | + | + | - | - | - | Dyspepsia | Lt. chest  (Physician) | Doxy(10) | 2 | 0 |
| 6 | 81/F | Dec-15 | UK | 10 | 38.2 | + | - | - | + | - | Poor oral intake,  Cold sweating | Lt. knee  (Physician) | Doxy(7) | 2 | 0 |
| 7 | 70/F | Jun-13 | 3 | 10 | 39.4 | + | + | - | - | + | Confused mental state | Buttock  (Physician) | Cef(1)→Doxy(4) | 3 | 0 |
|  |  |  |  |  |  |  |  |  |  |  |  |  |  |  |  |
| 8 | 53/F | Apr-17 | 13 | NA | - | + | - | + | - | - | Febrile sensation | Lt. forearm  (Patient) | Doxy(5) | - | - |
| 9 | 80/F | May-17 | 7 | NA | 37.8 | + | + | + | - | + | Febrile sensation | Lt. forearm  (Patient) | Doxy(7) | 5 | 0 |
| 10 | 75/F | May-17 | UK | 16 | 38.3 | - | - | - | - | + | Abnormal behavior,  Dyspepsia | Post. neck  (Physician) | Doxy(5) | - | 0 |
| 11 | 81/F | May-17 | NA | 13 | 39.6 | + | + | + | - | - | Drowsy mental state  Febrile sensation | Rt. ant. neck  (Physician) | Doxy(11) →Mero(25) | 23 | 4.6 days† |
|  |  |  |  |  |  |  |  |  |  |  |  |  |  |  |  |
| 12 | 82/F | Jul-17 | 20 | 11 | - | - | - | - | - | + | - | Rt. leg  (Patient) | Cef+Doxy(6) | - | - |
| 13 | 81/M | May-18 | 7 | 8 | 38.7 | - | - | - | - | - | General weakness  Febrile sensation | Rt. shoulder  (Patient) | Cef(6) | 0 | 4 |
| 14 | 71/M | Jun-18 | 7 | 10 | 38.2 | - | - | - | + | - | Hematuria, Chest discomfort | Lt. wrist  (Physician) | Doxy(15) | 2 | 15 |
| 15 | 49/M | Jun-18 | 6 | NA | 38.6 | + | - | + | - | - | Headache  Febrile sensation | Rt. back area  (Patient) | Doxy(6) | - | 12 |
| 16 | 55/F | Jun-18 | UK | NA | - | - | - | - | - | - | - | Rt. subclavian  (Patient) | Doxy(7) | - | - |
| 17 | 83/M | Jul-18 | 23 | 8 | 38.2 | - | - | - | + | + | Mental change  Febrile sensation | Rt. knee  (Patient) | Cef(2)+Doxy(7) | 4 | 24 |
| 18 | 51/F | Jun-18 | UK | NA | - | + | + | + | - | - | Headache | Lt. thigh  (Physician) | Cef+Doxy(10) | - | - |
| 19 | 56/F | Aug-18 | UK | 7 | - | + | - | + | - | - | - | Rt. foot  (Patient) | Cef(1)  →Doxy(14) | - | - |
| 20 | 68/M | Sep-18 | 4 | NA | - | - | - | - | - | + | Febrile sensation | - | Doxy(10) | - | - |
| 21 | 79/F | Sep-18 | UK | 16 | - | + | - | - | - | - | Febrile sensation | Rt. breast  (Physician) | Cef+Doxy(6)  →Doxy(3) | - | - |

**Abbreviations: NA, not available; N/V, nausea and vomiting; Cef, ceftriaxone; Doxy, doxycycline; Fluco, fluconazole; Mero, meropenem; UK, unknown; Rt, right; Lt, left; ant, anterior; Post, posterior.**

**Duration 1^¶^ is the days from the symptom onset to the initial visit(days)**

**Duration 2^*^ is the febrile days after admission(days)**

**Duration 3^†^ is the time to defervesce after doxycycline administration(hours or days)**

**In 4.6 days‡, fever subsided, but 4 days after, it recurred (38.5°C)**

**Supplementary Table 2. Laboratory findings and following antibody titer**

| No. | Symptom onset  date | Period  between symptom onset  and sampling | Antibiotics administration day  from  symptom onset | Laboratory finding | | | | | | | | PCR | | | | IFA | | | Morulae  (first/re-interpretation) |
| --- | --- | --- | --- | --- | --- | --- | --- | --- | --- | --- | --- | --- | --- | --- | --- | --- | --- | --- | --- |
|  |  |  |  | WBC (10^3^/µL)  (neutro/lymph)(%) | Platelet  (10^3^/µL) | CRP  (mg/dL) | AST  (U/L) | ALT  (U/L) | LDH  (U/L) | Procalcitonin  (ng/mL) | CPK  (U/L) | *16S*  *rRNA* gene | *groEL* gene | | *ankA*gene | | IgM | IgG |  |
| 1 | 6 | 6  24  39  71  99 | 6 | 3.31(91.9/2.2)  5.80(66.5/21.8)  9.31(83.8/8.4)  NA  NA | 24  259  174  NA  NA | 15.9  0.31  2.50  NA  NA | 58  28.3  22.1  NA  NA | 34  31.6  18.9  NA  NA | 619  NA  NA  NA  NA | 1.6  NA  NA  NA  NA | 309  NA  NA  NA  NA | +  +  -  -  - | +  +  -  -  - | | +  -  -  -  - | | <1:16  NA  <1:16  <1:16  <1:16 | <1:80  NA  1:80  1:320  1:640 | -/- |
| 2 | 10 | 10  23  37  65 | 10 | 2.45(62.4/23.6)  NA  5.72(44.1/43.8)  NA | 78  NA  322  NA | 7.42  NA  NA  NA | 87  NA  NA  NA | 53  NA  NA  NA | 901  NA  NA  NA | 1.6  NA  NA  NA | 190  NA  NA  NA | -  -  NA  NA | +  -  NA  NA | | -  -  NA  NA | | <1:16  <1:16  <1:16  <1:16 | 1:80  1:160  1:80  1:160 | -/- |
| 3 | 10 | 10  37 | 10 | 3.24(83/10.2)  NA | 45  NA | 14.1  NA | 112  NA | 81  NA | 895  NA | NA  NA | 232  NA | +  - | +  - | +  - | | 1:16  1:64 | | 1:80  1:640 | -/- |
| 4 | 9 | 9  15  21 | 9 | 4.50(90.9/8.5)  NA  6.68(51.6/37.2) | 35  NA  295 | 16.3  NA  NA | 114  NA  28.2 | 45  NA  19.1 | 970  NA  NA | 8.6  NA  NA | 173  NA  NA | +  +  NA | +  +  NA | +  +  NA | | <1:16  1:64  NA | | 1:160  1:5120  NA | -/+ |
| 5 | 3 | 3  12  19 | 4 | 1.99(79.7/11)  NA  NA | 39  NA  NA | 20.8  NA  NA | 149  NA  NA | 67  NA  NA | 1087  NA  NA | 2.0  NA  NA | 416  NA  NA | +  +  - | +  -  - | +  +  - | | <1:16  1:16  <1:16 | | <1:80  <1:80  1:80 | -/- |
| 6 | 4 | 4 | 5 | 1.26(40.6/31.4) | 75 | 5.78 | 109 | 64 | 677 | 1.8 | 519 | + | + | + | | <1:16 | | <1:80 | -/- |
| 7 | 7 | 7  15  1447 | 7 | 2.22(86.8/8.4)  　9.76(25.4/65.3)  NA | 37  176  NA | 22  NA  NA | 99  NA  NA | 55  NA  NA | 1052  NA  NA | 8.3  NA  NA | 1394  NA  NA | +  +  NA | +  +  NA | +  +  NA | | <1:16  1:64  <1:16 | | <1:80  1:320  <1:80 | -/NA |
| 8 | 4 | 4  8  14  21  42  74  102  217 | 5 | 2.87(38.6/45.7)  4.26(42.2/48.5)  4.93(39.1/45.4)  4.32(49.1/39.7)  4.27(54.9/35.1)  5.73(55.8/33.8)  5.43(81.7/14.2)  NA | 175  326  338  290  297  320  296  NA | 0.15  NA  NA  NA  NA  NA  NA  NA | 84  39.8  15.2  14.1  14.5  15.3  19.8  41.6 | 75  60.4  16.7  11.5  11.3  16.4  14.9  32.6 | NA  NA  NA  NA  NA  NA  NA  NA | 0.09  NA  NA  NA  NA  NA  NA  NA | 59  NA  NA  NA  NA  NA  NA  NA | +  NA  NA  NA  NA  NA  NA  NA | -  　NA  NA  NA  NA  NA  NA  NA | -  NA  NA  NA  NA  NA  NA  NA | | NA  1:16  1:64  1:64  1:128  1:128  1:256  1:64 | | NA  <1:80  <1:80  1:80  1:80  <1:80  <1:80  <1:80 | -/+ |
| 9 | 3 | 3  7  10  17 | 3 | 2.67(66.7/18.4)  7.19(35.9/47.5)  6.10(50.9/34.5)  4.36(50.6/38.0) | 49  202  299  347 | 8.48  1.82  0.71  NA | 65  78.5  59.3  25.8 | 25  37.2  34.4  13 | NA  NA  NA  NA | 4.53  NA  NA  NA | 58  NA  NA  NA | +  +  NA  NA | +  +  　NA  　NA | +  +  NA  NA | | 1:256  1:512  1:512  1:64 | | <1:80  1:80  1:320  1:320 | -/- |
| 10 | 5 | 5  9  11 | 5 | 3.28(88.8/7.2)  　2.04(55.7/22.7)  7.53(51.8/32.6) | 86  64  341 | 28.6  28.6  NA | 85  98.2  26.7 | 65  71.1  32.5 | NA  NA  NA | 0.59  NA  NA | 99  309  NA | +  NA  + | +  　NA  　+ | +  NA  + | | 1:512  NA  1:1024 | | <1:80  NA  <1:80 | -/- |
| 11 | 6 | 6  12  27  33  41  57  72 | 6 | 9.26(91.6/5.6)  9.44(89.1/6.4)  18.54(94.7/3.5)  　7.24(78.6/13.7)  6.64(64.2/27.3)  NA  7.52(52.6/38.8) | 72  183  610  547  475  NA  302 | 31.4  16.9  NA  9.16  7.57  NA  NA | 159  44  NA  25.1  32.3  NA  19.2 | 47  20.4  NA  13.6  13.2  　NA  9.0 | NA  NA  NA  NA  NA  NA  NA | 5.52  1.12  0.142  NA  NA  NA  NA | 255  NA  NA  NA  NA  NA  NA | +  NA  NA  NA  NA  NA  NA | +  NA  NA  NA  NA  NA  NA | +  NA  NA  NA  NA  NA  NA | | <1:16  1:64  1:64  1:256  1:64  1:512  1:256 | | <1:80  1:160  1:640  1:640  1:640  1:640  1:640 | +/+ |
| 12 | 7 | 7  15  29 | 7 | 5.82(78.9/10.4)  5.18(53.2/30.9)  5.02(49.4/39.8) | 59  375  229 | 11.3  　NA  　NA | 47  27.8  NA | 28  29  NA | NA  NA  NA | 2.93  NA  NA | 413  NA  NA | +  　-  NA | +  　 -  　NA | +  　-  NA | | 1:256  1:1024  1:2048 | | <1:80  1:1280  1:2560 | NA/NA |
| 13 | 3 | 3  12  16  26  36 | 6 | 2.06(76.1/16.1)  4.73(64.4/24.3)  5.67(66.4/27.2)  NA  7.34(52.0/39.0) | 112  381  196  NA  265 | 3.60  0.40  4.92  NA  NA | 625.8  73  75.2  NA  39.7 | 133.9  51.4  33.2  NA  14.1 | NA  NA  NA  NA  NA | 0.473  NA  0.497  NA  NA | 18354  293  243  NA  NA | +  +  +  -  NA | +  +  +  -  NA | +  +  +  -  NA | | <1:16  1:1024  1:2048  >1:2048  1:2048 | | <1:80  1:80  1:320  >1:1280  1:20480 | -/- |
| 14 | 3 | 3  9  14  20  28 | 3 | 4.54(82.0/8.8)  4.30(45.8/43.7)  5.01(70.0/19.0)  6.14(65.4/23.9)  NA | 86  164  204  172  NA | 11.85  NA  0.70  NA  NA | 58.1  NA  36.8  NA  NA | 28  NA  27.5  NA  NA | 441  NA  NA  NA  NA | 2.46  NA  NA  NA  NA | 113  NA  NA  NA  NA | +  -  NA  NA  NA | +  +  NA  NA  NA | +  +  NA  NA  NA | | <1:16  <1:16  <1:16  <1:16  <1:16 | | <1:80  <1:80  1:160  1:1280  1:640 | -/+ |
| 15 | 6 | 6  20 | 6 | 4.18(73.7/15.3)  6.17(41.8/49.1) | 167  368 | 4.72  NA | 25  18.7 | 23.7  19.7 | NA  NA | -  NA | 145  NA | +  NA | +  NA | +  NA | | <1:16  1:128 | | <1:80  1:320 | -/- |
| 16 | 18 | 18  21  27 | 21 | NA  4.59(61.8/23.3)  5.85(50.0/42.4) | NA  198  221 | 0.08  0.17  NA | 17.9  18.9  NA | 17.1  18.9  NA | NA  NA  NA | NA  NA  NA | NA  NA  NA | -  NA  NA | +  NA  NA | +  NA  NA | | <1:16  1:16  1:32 | | <1:80  <1:80  <1:80 | -/- |
| 17 | 30 | 30  34  37  47  72 | 30 | 2.29(80.9/12.2)  4.59(42.0/41.0)  6.57(36.4/55.9)  6.67945.9/44.1)  7.60(46.4/42.8) | 113  95  178  184  153 | 4.94  3.06  0.59  NA  NA | 32  32.1  50.3  NA  17.3 | 12.4  18.7  34.0  NA  13.7 | 383  NA  NA  NA  NA | NA  NA  NA  NA  NA | NA  NA  NA  NA  NA | +  +  -  NA  NA | +  +  +  NA  NA | +  +  +  NA  NA | | 1:16  <1:16  1:16  1:128  1:128 | | <1:80  <1:80  <1:80  1:320  1:640 | -/- |
| 18 | 2 | 2  12  14  36 | 2 | 8.32(59.3/32.0)  7.08(42.9/49.0)  NA  7.80(32.3/60.0) | 123  264  NA  228 | NA  0.11  NA  NA | 144.3  24.2  NA  17.9 | 382.1  51.4  NA  28.0 | NA  NA  NA  NA | NA  NA  NA  NA | 69  NA  NA  NA | -  NA  NA  NA | -  NA  NA  NA | +  NA  NA  NA | | <1:16  <1:16  <1:16  <1:16 | | 1:320  1:320  1:640  1:640 | -/- |
| 19 | 3 | 3  5  19 | 3 | 3.06(83.0/13.4)  2.66(11/60)  5.74(34.6/54.2) | 123  111  257 | 2.39  NA  NA | 29.3  NA  NA | 24.8  NA  NA | NA  495  NA | 0.16  NA  NA | 183  NA  NA | +  NA  NA | +  NA  NA | +  NA  NA | | <1:16  <1:16  1:1024 | | 1:80  1:80  1:640 | NA/NA |
| 20 | 6 | 6  11  25  38 | 6 | 4.34(36/40)  9.01(39.9/48.7)  5.92(37.5/47.3)  NA | 114  306  223  NA | 3.53  NA  NA  NA | 231  33.2  19.6  NA | 281  126  23.6  NA | NA  469  NA  NA | NA  NA  NA  NA | NA  60  NA  NA | +  NA  NA  NA | +  NA  NA  NA | +  NA  NA  NA | | 1:16  1:256  NA  1:2048 | | <1:80  1:160  NA  1:160 | NA/NA |
| 21 | 3 | 3  10  17 | 3 | 2.00(77.0/19.0)  10.40(18.2/73.9)  5.96(16.6/73.0) | 63  214  433 | 4.74  0.50  NA | 152.5  46.3  NA | 101.5  25.3  NA | NA  NA  NA | 0.494  NA  NA | 40  NA  NA | +  NA  NA | +  NA  NA | +  NA  NA | | <1:16  1:16  1:256 | | <1:80  1:160  1:1280 | -/- |

**Abbreviations: neutron, neutrophil; lymph, lymphocyte; PCR, polymerase chain reaction; IFA, indirect immunofluorescence antibody assay; CRP, C-reactive protein; AST, aspartate aminotransferase; ALT, alanine aminotransferase; CPK, creatine phosphokinase; LDH, lactate dehydrogenase; NA, not available.**

**Supplementary Table 3. Changes in laboratory findings by sampling date**

| **Variable**  **(Normal range)** | **Admission date** | | **1 week from admission** | | **2 weeks from admission** | | **P-value*** |
| --- | --- | --- | --- | --- | --- | --- | --- |
|  | **N** | **Median (IQR)** | **N** | **Median (IQR)** | **N** | **Median (IQR)** |  |
| **WBC (/mm^3^)**  **(4,000–10,800)** | 20 | 3912  (2505–4460) | 20 | 7442  (5092–8835) | 14 | 5692.9  (4350–6550) | 0.002 |
| **Neutrophil (%)**  **(40–80)** | 21 | 67.5  (60 –88) | 20 | 42.6  (33.5–55.8) | 14 | 48.9  (40.3–63.5) | 0.002 |
| **Lymphocyte (%)**  **(25–50)** | 20 | 14.6  (6.3–18.8) | 20 | 46.1  (33.5–55.8) | 14 | 40.36  (26.8–50.3) | <0.001 |
| **PLT (×10^3^/mm^3^)**  **(150–400)** | 20 | 85.6  (50.3–114) | 20 | 255.6  (184–330) | 14 | 289.5  (220–352.2) | <0.001 |
| **CRP (mg/dL)**  **(0.0–0.3)** | 12 | 9.0  (4.0–13.5) | 11 | 1.0  (1.0–1.0) | 2 | 11  (5–11.0) | NA |
| **AST (U/L)**  **(5–40)** | 21 | 110.3  (50.5–128) | 20 | 62.8  (28–60.5) | 10 | 30.2  (22.8–30.5) | <0.001 |
| **ALT (U/L)**  **(5–40)** | 21 | 75.2  (24.5–77) | 20 | 59.8  (29–57) | 10 | 21.0  (14.5–26.8) | 0.001 |
| **LDH (U/L)**  **(200–450)** | 5 | 854.8  (695.5–994) | 2 | 488  (469–488) | 0 | NA | NA |
| **Procalcitonin (ng/mL)**  **(0–0.5)** | 12 | 4  (2.0–5.0) | 0 | NA | 0 | NA | NA |
| **CPK (U/L)**  **(55–215)** | 19 | 9924.7  (99–413) | 3 | 345.3  (30–175) | 1 | 243 | NA |

**Abbreviations: PLT, platelet; CRP, C-reactive protein; AST, aspartate aminotransferase; ALT, alanine aminotransferase; CPK, creatine phosphokinase; LDH, lactate dehydrogenase; NA, not available; IQR, interquartile range.**

**^*^Friedman test for differences in laboratory findings by sampling date**

**Supplementary Table 4. Anaplasmosis IFA IgM and IgG titers by time after the onset of symptoms**

| **IFA** | |  | | **1 week** | | | **2 weeks** | | | | **3 weeks** | | | | **4 weeks*** | | | | **3 M^†^** | | | | **6 M^‡^** | | | | **1 Y** | | | **4 Y** | | | | | **5 Y** | | | | |
| --- | --- | --- | --- | --- | --- | --- | --- | --- | --- | --- | --- | --- | --- | --- | --- | --- | --- | --- | --- | --- | --- | --- | --- | --- | --- | --- | --- | --- | --- | --- | --- | --- | --- | --- | --- | --- | --- | --- | --- |
| IgM  titer | |  | | No | CP | | No | | CP | | No | | CP | | No | | CP | | No | CP | | | No | | CP | | No | | CP | No | | | CP | | No | | CP | | |
| 0 | |  | | 10 | 100 | | 3 | | 100 | | 3 | | 100 | | 3 | | 100 | | 2 | 100 | | | 0 | | 100 | | 1 | | 100 | 1 | | | 100 | | 1 | | 100 | | |
| **1:16** | |  | | **1** | **23.1** | | **5** | | **78.6** | | **0** | | **72.7** | | **1** | | **75** | | **0** | **66.7** | | | **0** | | **100** | | **0** | | **0** | **0** | | | **0** | | **0** | | **0** | | |
| 1:32 | |  | | 0 | 15.4 | | 0 | | 42.9 | | 1 | | 72.7 | | 0 | | 66.7 | | 0 | 66.7 | | | 0 | | 100 | | 0 | | 0 | 0 | | | 0 | | 0 | | 0 | | |
| 1:64 | |  | | 0 | 15.4 | | 1 | | 42.9 | | **4** | | 63.6 | | 2 | | 66.7 | | 0 | 66.7 | | | 1 | | 100 | | 0 | | 0 | 0 | | | 0 | | 0 | | 0 | | |
| 1:128 | |  | | 0 | 15.4 | | 0 | | 35.7 | | 1 | | 27.3 | | 2 | | 50 | | 1 | 66.7 | | | 1 | | 50 | | 0 | | 0 | 0 | | | 0 | | 0 | | 0 | | |
| 1:256 | |  | | 0 | 15.4 | | 2 | | 35.7 | | 0 | | 18.2 | | 1 | | 33.3 | | 2 | 50 | | | 0 | | 0 | | 0 | | 0 | 0 | | | 0 | | 0 | | 0 | | |
| 1:512 | |  | | 2 | 15.4 | | 1 | | 21.4 | | 0 | | 18.2 | | 0 | | 25 | | 1 | 16.7 | | | 0 | | 0 | | 0 | | 0 | 0 | | | 0 | | 0 | | 0 | | |
| 1:1024 | |  | | 0 | 0 | | 2 | | 14.3 | | 1 | | 18.2 | | 0 | | 25 | | 0 | 0 | | | 0 | | 0 | | 0 | | 0 | 0 | | | 0 | | 0 | | 0 | | |
| 1:2048 | |  | | 0 | 0 | | 0 | | 0 | | 1 | | 9.1 | | 2 | | 25 | | 0 | 0 | | | 0 | | 0 | | 0 | | 0 | 0 | | | 0 | | 0 | | 0 | | |
| 1:4096 | |  | | 0 | 0 | | 0 | | 0 | | 0 | | 0 | | 1 | | 8.3 | | 0 | 0 | | | 0 | | 0 | | 0 | | 0 | 0 | | | 0 | | 0 | | 0 | | |
| Total | |  | | 13 |  | | 14 | |  | | 11 | |  | | 12 | |  | | 6 |  | | | 2 | |  | | 1 | |  | 1 | | |  | | 1 | |  | | |
|  | |  | |  |  | |  | |  | |  | |  | |  | |  | |  |  | | |  | |  | |  | |  |  | | |  | |  | |  | | |
| **IFA** |  | | **1 week** | | | **2 weeks** | | | | **3 weeks** | | | | **4 weeks*** | | | | **3 M^†^** | | | | **6 M^‡^** | | | | **1 Y** | | | | | **4 Y** | | | **5 Y** | | | | |  |
| IgG  titer |  | | No | | CP | No | | CP | | No | | CP | | No | | CP | | No | | | CP | No | | CP | | No | | CP | | | No | CP | | No | | CP | |  |  |
| 0 |  | | 11 | | 100 | 5 | | 100 | | 1 | | 100 | | 1 | | 100 | | 2 | | | 100 | 2 | | 100 | | 1 | | 100 | | | 1 | 100 | | 1 | | 100 | |  |  |
| **1:80** |  | | **2** | | **15.4** | **2** | | **64.3** | | **2** | | **91** | | **3** | | **91.7** | | **1** | | | **66.7** | **0** | | **0** | | **0** | | **0** | | | **0** | **0** | | **0** | | **0** | |  |  |
| 1:160 |  | | 0 | | 0 | 5 | | 50 | | 0 | | 72.7 | | 0 | | 66.7 | | 1 | | | 50 | 0 | | 0 | | 0 | | 0 | | | 0 | 0 | | 0 | | 0 | |  |  |
| 1:320 |  | | 0 | | 0 | 2 | | 14.3 | | 4 | | 72.7 | | 1 | | 66.7 | | 1 | | | 33.3 | 0 | | 0 | | 0 | | 0 | | | 0 | 0 | | 0 | | 0 | |  |  |
| 1:640 |  | | 0 | | 0 | 0 | | 0 | | 2 | | 36.4 | | 4 | | 58.3 | | 1 | | | 16.7 | 0 | | 0 | | 0 | | 0 | | | 0 | 0 | | 0 | | 0 | |  |  |
| 1:1280 |  | | 0 | | 0 | 0 | | 0 | | 1 | | 18.2 | | 0 | | 25 | | 0 | | | 0 | 0 | | 0 | | 0 | | 0 | | | 0 | 0 | | 0 | | 0 | |  |  |
| 1:2560 |  | | 0 | | 0 | 0 | | 0 | | 0 | | 9.1 | | 2 | | 25 | | 0 | | | 0 | 0 | | 0 | | 0 | | 0 | | | 0 | 0 | | 0 | | 0 | |  |  |
| 1:20480 |  | | 0 | | 0 | 0 | | 0 | | 1 | | 0 | | 1 | | 8.3 | | 0 | | | 0 | 0 | | 0 | | 0 | | 0 | | | 0 | 0 | | 0 | | 0 | |  |  |
| Total |  | | 13 | |  | 14 | |  | | 11 | |  | | 12 | |  | | 6 | | |  | 2 | |  | | 1 | |  | | | 1 |  | | 1 | |  | |  |  |

**Abbreviations: IFA, indirect immunofluorescent assay; CP, cumulative percentage; M, month; Y, year; No, number.**

**4 weeks^*^includes from 4–7 weeks.**

**3 M^†^ includes from 2–4 months.**

**6 M^‡^ includes from 5– 9 months.**
